# Microfluidic PCR Amplification and MiSeq Amplicon Sequencing Techniques for High-Throughput Detection and Genotyping of Human Pathogenic RNA Viruses in Human Feces, Sewage, and Oysters

**DOI:** 10.3389/fmicb.2018.00830

**Published:** 2018-04-27

**Authors:** Mamoru Oshiki, Takayuki Miura, Shinobu Kazama, Takahiro Segawa, Satoshi Ishii, Masashi Hatamoto, Takashi Yamaguchi, Kengo Kubota, Akinori Iguchi, Tadashi Tagawa, Tsutomu Okubo, Shigeki Uemura, Hideki Harada, Naohiro Kobayashi, Nobuo Araki, Daisuke Sano

**Affiliations:** ^1^Department of Civil Engineering, National Institute of Technology, Nagaoka, Japan; ^2^Department of Environmental Health, National Institute of Public Health, Wako, Japan; ^3^Center for Simulation Sciences and Informational Biology, Ochanomizu University, Bunkyô, Japan; ^4^Center for Life Science Research, University of Yamanashi, Kofu, Japan; ^5^Department of Soil, Water and Climate, University of Minnesota, Minneapolis, MN, United States; ^6^Department of Environmental Systems Engineering, Nagaoka University of Technology, Nagaoka, Japan; ^7^Department of Science of Technology Innovation, Nagaoka University of Technology, Nagaoka, Japan; ^8^Department of Civil and Environmental Engineering, Tohoku University, Sendai, Japan; ^9^Faculty of Applied Life Sciences, Niigata University of Pharmacy and Applied Life Sciences, Niigata, Japan; ^10^New Industry Creation Hatchery Center, Tohoku University, Sendai, Japan

**Keywords:** human pathogenic viruses, high-throughput detection and genotyping, microfluidic PCR, MiSeq sequencing, sewage, human feces, oyster

## Abstract

Detection and genotyping of pathogenic RNA viruses in human and environmental samples are useful for monitoring the circulation and prevalence of these pathogens, whereas a conventional PCR assay followed by Sanger sequencing is time-consuming and laborious. The present study aimed to develop a high-throughput detection-and-genotyping tool for 11 human RNA viruses [Aichi virus; astrovirus; enterovirus; norovirus genogroup I (GI), GII, and GIV; hepatitis A virus; hepatitis E virus; rotavirus; sapovirus; and human parechovirus] using a microfluidic device and next-generation sequencer. Microfluidic nested PCR was carried out on a 48.48 Access Array chip, and the amplicons were recovered and used for MiSeq sequencing (Illumina, Tokyo, Japan); genotyping was conducted by homology searching and phylogenetic analysis of the obtained sequence reads. The detection limit of the 11 tested viruses ranged from 10^0^ to 10^3^ copies/μL in cDNA sample, corresponding to 10^1^–10^4^ copies/mL-sewage, 10^5^–10^8^ copies/g-human feces, and 10^2^–10^5^ copies/g-digestive tissues of oyster. The developed assay was successfully applied for simultaneous detection and genotyping of RNA viruses to samples of human feces, sewage, and artificially contaminated oysters. Microfluidic nested PCR followed by MiSeq sequencing enables efficient tracking of the fate of multiple RNA viruses in various environments, which is essential for a better understanding of the circulation of human pathogenic RNA viruses in the human population.

## Introduction

Various species of human RNA viruses, including Aichi virus (AiV), astrovirus (AstV), enterovirus (EV), hepatitis A virus (HAV), hepatitis E virus (HEV), norovirus (NoV), rotavirus (RV), sapovirus (SaV), and human parechovirus (HPeV), have been recognized as causative agents of waterborne viral gastroenteritis and hepatitis (Bosch, 1998; Harvala et al., 2008; Sinclair et al., 2009; Pham et al., 2010; Svraka et al., 2010; Drexler et al., 2011). The above mentioned RNA virus species includes multiple (sub)genogroups. For example, NoV is composed of seven genogroups, and each genogroup is further subdivided into genotypes (Vinjé, 2015). These RNA viruses circulate in aquatic environments and the human population (Iwai et al., 2009; Sano et al., 2011; Hata et al., 2015; Miura et al., 2016; Pu et al., 2016). For instance, the viruses exist at high concentration in feces of patients and are released into sewage system (Lee et al., 2007; Drexler et al., 2011). Sewage effluents still contain the viruses at certain level (Miura et al., 2015; Kobayashi et al., 2017), which contaminate natural water resources. The released viruses can be accumulated in shellfishes such as oyster, and outbreak occurs by eating the contaminated shellfish (Le Guyader et al., 1994; Ueki et al., 2005; Nenonen et al., 2008). For a better understanding of the circulation and prevalence of RNA viruses and for identification of a contamination source, detection and genotyping of multiple viruses are essential (Kazama et al., 2017).

Detection and genotyping of human pathogenic RNA viruses have usually been carried out by reverse transcription (RT)-PCR followed by Sanger sequencing (Nasri et al., 2007; Stene-Johansen et al., 2007; Kitajima et al., 2010) because of the high sensitivity, specificity, and reliability of identification of contaminating viruses. Nonetheless, clinical samples (e.g., stool samples) (Román et al., 2003; Iturriza Gómara et al., 2008) and environmental water samples such as river water (Ishii et al., 2014) and wastewater (Miura et al., 2015; Kobayashi et al., 2017) contain various species of human pathogenic RNA viruses, and the conventional PCR assay is labor intensive because many PCR runs are required for detection and genotyping of multiple RNA viruses. Additionally, nested PCR in which the amplicon from the first round of PCR undergoes the second round PCR as a template DNA is often conducted to enhance sensitivity and specificity of detection and genotyping of an RNA virus (Le Guyader et al., 1994; Fischer et al., 2003; Aw et al., 2009); this approach further increases the amount of labor. Moreover, recombination of viral genomes has resulted in emergence of viral variants containing a genome deriving from different genotypes (Eden et al., 2013). As for NoV GII, new GII genotype 2 (GII.2) epidemic strains containing open-reading frame 1 (ORF1) and ORF 2–3 regions of GII.5 and GII.2, respectively, were recently identified (Motomura et al., 2016). In this case, the use of multiple oligonucleotide primer sets targeting different regions of the viral genome is recommended for reliable genotyping, while this practice is even more laborious.

Apart from the PCR-based assay, shotgun sequencing analysis using a next-generation sequencer (NGS) was recently developed for the detection and identification of a human pathogenic RNA virus in human feces and wastewater samples (Batty et al., 2013; Hasing et al., 2016). Abundance of the sequence reads corresponding to NoV in stool samples was, however, only 0.01–1.9% of all sequence reads (Hasing et al., 2016), requiring massive sequencing efforts for detection of the human pathogenic RNA viral population in the sample. This situation increases labor and sequencing costs; therefore, only a limited number of samples can be analyzed this way.

The present authors have developed high-throughput quantitative PCR (qPCR) techniques involving a microfluidic chip for detection and quantification of pathogenic microbes (Ishii et al., 2013, 2014). In the previous microfluidic qPCR (MFqPCR) analyses, which used a 96.96 Dynamic Array chip (Fluidigm, Tokyo, Japan), up to 9,216 independent PCRs could be carried out simultaneously in nanoliter reaction chambers on a microfluidic chip, and the MFqPCR enables simultaneous quantification of 13 viruses in up to 92 samples by means of 13 sets of oligonucleotide primers and TaqMan probes: i.e., adenovirus as a DNA virus and AiV, AstV, EV, NoV GI, NoV GII, NoV GIV, HAV, HEV, RV, SaV, mengovirus, and murine NoV as RNA viruses. Sensitivity and specificity of MFqPCR are comparable with those of a conventional qPCR assay (Ishii et al., 2014), and the MFqPCR has provided a convenient and efficient platform for PCR detection of RNA viruses in water environments (Kobayashi et al., 2017). Moreover, the use of a different type of microfluidic chip, specially designed for the recovery of PCR amplicons (i.e., a 48.48 access array chip, Fluidigm, Tokyo, Japan), allows for the recovery of amplicons after microfluidic PCR, and for their subsequent sequencing using NGS. This approach has been successfully applied in human leukocyte antigen typing (Lange et al., 2014), in genotyping of enterohemorrhagic *Escherichia coli* (Ison et al., 2016), and in the investigation of the genetic diversity of multiple functional genes related to the nitrogen cycle (Oshiki et al., 2018), but has not yet been used for the genotyping of human pathogenic viruses. The use of microfluidic PCR in combination with amplicon sequencing is a promising approach for simultaneous high-throughput detection and genotyping of multiple viral species in multiple samples, the performance of which (in terms of sensitivity, specificity, and applicability to different types of samples) remains to be investigated.

**FIGURE 1:**
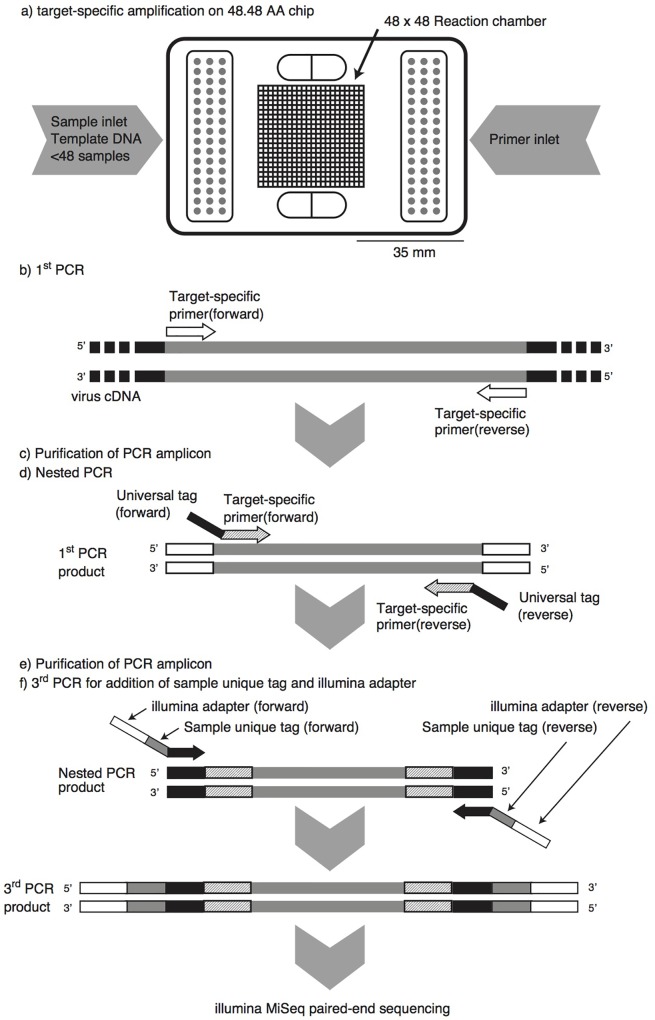
Pipeline of microfluidic nested PCR followed by MiSeq amplicon sequencing (MFnPCR–MiSeq). Purification of PCR amplicons was carried out with the NucleoSpin Gel and PCR Clean-up Kit. Amplification by the first-round and nested PCR was performed on a 48.48 Access Array (AA) chip in a BioMark HD reader, while the third PCR for addition of a sample-specific tag and Illumina adapter was run in 96-well PCR plates on a conventional thermal cycler.

Accordingly, the aim of this study was to develop a high-throughput tool for detection and genotyping of 11 human pathogenic RNA viruses by microfluidic nested PCR followed by MiSeq amplicon sequencing (MFnPCR–MiSeq; **Figure 1**). The following 11 human pathogenic RNA viruses were targeted in this study: AiV, AstV, EV, NoV GI, NoV GII, NoV GIV, HAV, HEV, RV, SaV, and HPeV. Notably, the oligonucleotide primers used in our previous MFqPCR assay (Ishii et al., 2014) could not be used to analyze the 11 human pathogenic RNA viruses listed above, as amplicon sequence lengths (ranging from 87 to 172 bp, 113 bp on average) were too short to allow for reliable genotyping. Therefore, 34 sets of newly selected oligonucleotide primers (Supplementary Table S1) were used in the present study. After validation of sensitivity and specificity, MFnPCR–MiSeq was applied to sewage and human fecal samples collected from patients. To the best of our knowledge, this is the first study combining microfluidic PCR and next-generation sequencing techniques for detection and genotyping of multiple human RNA viruses in environments.

## Materials and Methods

### Standard DNA Samples

Sensitivity of MFnPCR–MiSeq was examined in a mixture of plasmid DNA and viral cDNA (**Table 1**). As for NoV GI, NoV GII, NoV GIV, HAV, HEV, and HPeV, plasmids containing the target region were constructed or prepared as previously described (Ishii et al., 2014). The target regions were obtained from the following reference strains: Norwalk (nucleotide region 5271–5770), Lordsdale (4998–5497), Saint-Cloud (681–1180), HM175 (2151–3440), Burma (5147–6646), and Harris (2101–3250). DNA concentrations were determined by means of the Qubit dsDNA BR Assay Kit on a Qubit 3.0 Fluorometer (Thermo Fisher Scientific K.K., Yokohama, Japan). As for AiV, AstV, EV, RV, and SaV, viral RNA was extracted from cell culture or fecal samples, cDNA was synthesized, and copy numbers were determined by conventional qPCR (Ishii et al., 2014). The cDNA of AiV strain N1277/91 was kindly provided by Dr. Hiroyuki Katayama (The University of Tokyo, Japan). Poliovirus 1 strain Sabin is courtesy of Dr. Kazuyoshi Yano (Tokyo Metropolitan Institute of Public Health, Japan) and served as the reference strain of EV. RV strain RRV was kindly provided by Prof. Osamu Nakagomi (Nagasaki University, Japan). AstV and SaV were obtained from feces and were a generous gift from Dr. You Ueki (Miyagi Prefectural Institute of Public Health and Environment, Japan). The six plasmids and five synthesized cDNAs were mixed at equimolar concentrations and serially diluted from 10^0^ to 10^4^ copies/μL.

**Table 1:** Plasmid DNAs and viral cDNAs serving as positive controls for detection by microfluidic nested PCR followed by MiSeq amplicon sequencing.

Virus	Template	Genotype/serotype	Strain	Accession no.
NoV GI	Plasmid	GI.1	Norwalk	M87661
NoV GII		GII.4	Lordsdale	X86557
NoV GIV		GIV.1	Saint-Cloud	AF414427
HAV		IB	HM-175	M14707
HEV		1	Burma	M73218
HPeV		1	Harris	S45208
AiV	cDNA	B	N1277/91	–
EV		Poliovirus 1	Sabin	–
RV		G3P[3]	RRV	–
AstV		HAstV-1	13K0603	–
SaV		GI.1	15K0402	–

### Contaminated Oysters

Live oysters were purchased directly from a producer in Miyagi Prefecture, Japan, and were artificially contaminated by placing in seawater seeded with a known concentration of a fecal suspension containing NoV GII genotype 4 (GII.4) Sydney 2012 or GII.17 Kawasaki 2014 strain (10^7^ copies/mL) for 24 h with aeration. Digestive tissues were excised from 15 contaminated oysters and chopped up to obtain a homogenate. Viral RNA was extracted from 2 g of the digestive-tissue homogenate according to the method from ISO with some modifications. Briefly, 100 μL of the murine NoV process control and 2 mL of a 100 μg/mL proteinase K solution (Cat. No. P2308, Sigma-Aldrich, Tokyo, Japan) were added to the 2 g of the digestive-tissue homogenate. The sample was incubated at 60°C for 15 min on a rotator (32 rpm) and then centrifuged at 3,000 ×*g* for 5 min. Approximately 2.5 mL of the resulting supernatant was collected and 500 μL was used for RNA extraction with the NucliSENS Kit (bioMérieux, Tokyo, Japan).

### Human Fecal Samples

Eight fecal samples that have tested positive for NoV GI or GII were kindly provided by Dr. You Ueki (Miyagi Prefectural Institute of Public Health and Environment, Japan). The samples were collected from patients with acute gastroenteritis through routine infectious disease surveillance in Miyagi Prefecture, Japan, during the National Epidemiological Surveillance of Infectious Diseases from March 2014 to March 2015. General practitioners obtained oral consent for sample collection and pathogen screening from patients. Screening of pathogens was performed as a part of health services provided by Miyagi Prefectural Institute of Public Health and Environment. Consequently, human ethics approval and informed consent were not required for the present study. All samples were anonymized and there is no possibility of personal identification. The collected fecal samples were diluted in phosphate-buffered saline (pH 7) to obtain a 10% suspension and centrifuged at 10,000 ×*g* for 10 min. The resulting supernatants were stored at -80°C until processing, and viral RNA was extracted using the NucliSENS kit.

### Sewage Samples

These samples were collected at two domestic wastewater treatment plants in Niigata and Miyagi, Japan. Regarding the sewage samples collected from the wastewater treatment plant in Niigata (the population served by the sewerage system and daily flow rate, respectively: 150,000 people and 120,000 m^3^/d), the present authors have previously determined virus concentrations by MFqPCR, and these data are shown in Supplementary Table S2 (Kobayashi et al., 2017). The sewage samples were 24-h composite samples (1 L/h) that were collected from May 2014 to January 2015.

The other sewage sample was collected at a wastewater treatment plant (10,000 and 4,000 m^3^/d: population served by the sewerage system and daily flow rate, respectively) in Miyagi Prefecture in June 2015. One liter of the effluent of the first sedimentation tank was collected as a grab sample. The collected sewage sample was immediately transported to our laboratory on ice and subjected to concentration and recovery of virus particles by the polyethylene glycol precipitation method (Kazama et al., 2016). The virus concentrate was stored at -80°C until processing, and viral RNA was extracted by means of the QIAamp Viral RNA Mini Kit (Qiagen, Tokyo, Japan).

### cDNA Synthesis

This procedure was carried out using the PrimeScript RT Reagent Kit with a random hexamer primer (Takara Bio, Shiga, Japan), as previously described (Ishii et al., 2014).

### Oligonucleotide Primers

The 34 sets of oligonucleotide primers shown in Supplementary Table S1 were used in the present study for genotyping of the following 11 viruses: AiV, AstV, EV, HAV, HEV, NoV GI, NoV GII, NoV GIV, RV, SaV, and HPeV. Two primer sets were newly designed and employed in the present study for the detection of EV, i.e., primers EV1F_66 and EV1R_552 for the first round of PCR and primers EV1F-66 and Ev2R_460 for seminested PCR (see Supplementary Text). The forward and reverse PCR primers used for the nested PCR amplification contained the following Illumina tag sequences at the 5′-end: 5′-TCGTCGGCAGCGTCAGATGTGTATAAGAGACAG-3′ and 5′-GTCTCGTGGGCTCGGAGATGTGTATAAGAGACAG-3′, respectively. The sequence length of PCR amplicons without Illumina tag sequences ranges from 180 to 760 bp (341 bp on average).

### Microfluidic Nested PCR

Microfluidic PCR was run in triplicate on a BioMark HD reader with a 48.48 AccessArray chip (Fluidigm, Tokyo, Japan). The 20× primer solutions and presample master mix were dispensed into the 48.48 Access Array chip and mixed by an IFC controller AX (Fluidigm, Tokyo, Japan) according to the manufacturer’s instructions. The 20× primer solutions contained the 1× Access Array loading reagent (Fluidigm, Tokyo, Japan) and 4 μM each forward and reverse primers. The presample master mix (5 μL) contained 1× FastStart HiFi reaction buffer (Roche Diagnostics, Tokyo, Japan), 1× Access Array loading reagent, 4.5 mM MgCl_2_, 5% (v/v) dimethyl sulfoxide, 0.2 mM each dNTP, 0.05 U of FastStart HiFi Enzyme Blend (Roche Diagnostics), 1× EvaGreen (Biotium, CA, United States), 0.5× ROX, and 1 μL of template DNA (ca. 10 ng). Microfluidic PCR was performed under the following thermal conditions: 50°C for 120 s, 70°C for 20 min, 95°C for 10 min, followed by 10 cycles of program A (95°C for 15 s, 48°C for 30 s, and 72°C for 1 min), 2 cycles of program B (95°C for 15 s, 80°C for 30 s, 48°C for 30 s, and 72°C for 1 min), 8 cycles of program A, 2 cycles of program B, 10 cycles of program A, 2 cycles of program B, and the final extension at 72°C for 3 min. The detection of PCR products involved monitoring of the fluorescence intensity of the EvaGreen dye. The threshold cycle (*C*_T_) was defined as the cycle at which the EvaGreen signal became significantly greater than the background level. After the microfluidic PCR, the amplicons were recovered with the Access Array Harvest Reagent (Fluidigm, Tokyo, Japan) and the IFC controller AX.

For microfluidic nested-PCR amplification, the recovered 48 amplicons (approx. 10 μL) were purified using NucleoSpin Gel and the PCR Clean-up Kit (Takara Bio, Shiga, Japan). The purified DNAs were eluted with 15 μL of the supplied elution buffer. Microfluidic nested PCR was run with the purified DNAs as a template, and the amplicons were recovered from the chip as described above. The 48 nested-PCR products were purified by means of the NucleoSpin Gel and PCR Clean-up Kit and subjected to the following sequencing library preparation. A sample without viral cDNA (i.e., pure water) was also subjected to the above microfluidic first-round and nested PCR in parallel as a negative control.

### Sequencing Library Preparation and the MiSeq Run

The purified nested-PCR products were tagged with sample-specific index sequences at their 5’-end by PCR via the index primers supplied in Nextera XT index Kit v2 Set A (Illumina, Tokyo, Japan). The PCR mixture (20 μL) contained 1× KAPA HiFi HS ReadyMix (Nippon Genetics, Tokyo, Japan), 2 μL of forward and reverse index primers, 1 μL of the purified PCR products, and 5 μL of distilled water. The PCR was conducted under the following cycling conditions: 95°C for 3 min; 15 cycles of 95°C for 30 s, 55°C for 30 s, and 72°C for 1 min; and 72°C for 5 min. After agarose gel electrophoresis, gel lanes containing the amplicons of 200–1,000 bp were excised and purified using the NucleoSpin Gel and PCR Clean-up Kit. Tagged amplicons were pooled and analyzed in a MiSeq paired-end sequencing reaction with the v3 reagent kit (Illumina, Tokyo, Japan).

### Bioinformatic Analyses

These analyses were performed as follows in the MacQIIME 1.9.1 (Caporaso et al., 2010) and USEARCH 9.2 (Edgar, 2010) software packages. The merging of forward and reverse reads obtained from the MiSeq paired-end sequencing and the quality filtering of the merged reads were conducted via MacQIIME scripts: join_paired_ends.py and split_library.py with a minimum quality score parameter of 25 and a minimum sequence length of 70 bp.

The quality-filtered reads were demultiplexed by sample and clustered into operational taxonomic units (OTUs) by the usearch scripts. Specifically, the reads were dereplicated (-derep_fulllength –sizeout) and sorted, and singletons were removed (-sortbysize–minsize 2); then, the reads were clustered (-cluster_otus), checked for chimeric sequences (-uchime_denovo), and mapped back to OTUs at 97% identity (-usearch_global–strand both–id 0.97). When relative abundance of the OTU in total sequence reads of each sample was <0.1%, the OTU was eliminated and not subjected to the further analysis. The reads representing each OTU were subjected to a blastn search against the nt database, and virus reads were thus identified. For phylogenetic analysis, the virus reads were processed in ClustalW; bootstrapped phylogenetic trees were then constructed by the neighbor-joining method with 1,000 bootstrap replications in the MEGA7 software (Kumar et al., 2016). The genetic distances were calculated by the Kimura two-parameter method. Among NoV reads, genotypes and variants were identified using the NoV Genotyping Tool Version 1.0 (Kroneman et al., 2011) before the phylogenetic analysis.

### Nucleotide Sequence Accession Numbers

Nucleotide sequence data from the human feces, sewage, and contaminated oysters were deposited in the DDBJ/EMBL/GenBank databases under the accession number DRA005567.

## Results

### Determination of the Detection Limit on Positive Control Samples

**FIGURE 2:**
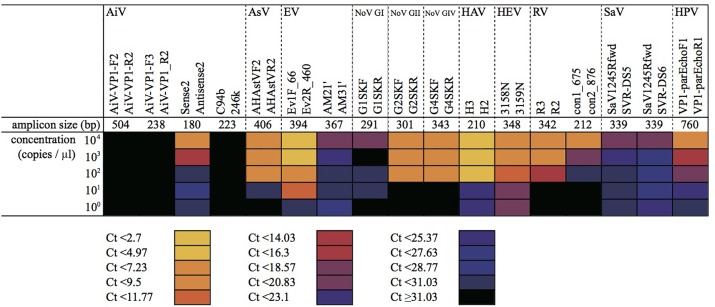
Detection of serially diluted virus samples by microfluidic nested PCR followed by MiSeq amplicon sequencing. Plasmid DNA and cDNA samples containing target regions of each virus were mixed at equimolar concentrations and diluted to 10^4^–10^0^ copies/μL. The mixtures were subjected to the first round of MF-PCR, and the amplicon was collected and used for MFnPCR with 20 oligonucleotide primer sets for detection of 11 viruses (i.e., AiV; AsV; EV; NoV GI, GII, and GIV; HAV; HEV; RV; SaV; and HPV). *C*_T_ values determined in the nested MF PCR are shown as a heat map. Amplicon size represents the size of a PCR amplicon without an Illumina tag sequence.

The mixture of plasmid DNA and viral cDNA was serially diluted from 10^4^ to 10^0^ copies/μL and then subjected to MFnPCR–MiSeq. As shown in **Figure 2**, all the tested viruses could be detected simultaneously when concentrations were higher than 10^3^ copies/μL. PCR amplicons obtained from each dilution series were recovered and subjected to MiSeq sequencing. Blast analysis of sequence reads revealed that the recovered amplicons derived from the plasmid DNA and viral cDNA serving as templates. When the detection limit was defined as 10 sequence reads per sample, each tested virus was detectable in the following dilution series (copies/μL): 10^2^ for AiV, 10^2^ for AstV, 10^1^ for EV, 10^1^ for NoV GI, 10^2^ for NoV GII, 10^2^ for NoV GIV, 10^1^ for HAV, 10^0^ for HEV, 10^2^ for RV, 10^3^ for SaV, and 10^2^ for HPeV. By considering dilution factors resulting from the processes of virus concentration, RNA extraction, and cDNA synthesis, the detection limits were calculated to be 10^1^–10^4^ copies/mL for sewage, 10^5^–10^8^ copies/g-human feces, and 10^2^–10^5^ copies/g-digestive tissues of oysters. Those detection limits were not dependent on the amplicon size (180–760 bp without Illumina tag sequences) (Supplementary Table S1) but on the PCR primer set. For instance, microfluidic nested PCR amplification was performed with four primer sets for detection of AiV, and PCR amplification was detectable only in the assay containing primers Sense 1 (6261) and 6779 in the first-round PCR and primers Sense 2 and Antisense 2 in the nested PCR. Likewise, multiple PCR primer sets were used for the detection of EV, RV, and SaV. The following PCR primer sets gave the highest sensitivity: Ev1F_66 and Ev2R_460 for EV; R3 and R2 for RV; and SaV1245Rfwd and SVR-DS5 for SaV. This means that the amplification efficiency of each primer set is more critical than amplicon size for the detection of target viruses.

**FIGURE 3:**
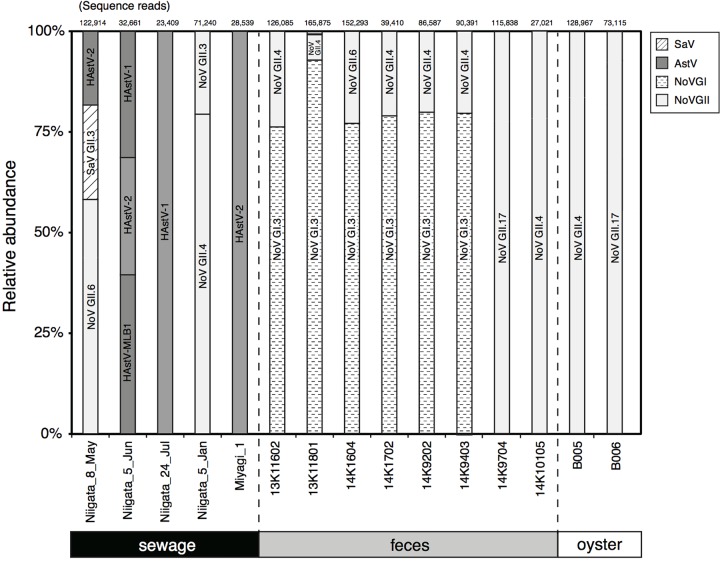
Detection and typing of viruses in samples of human feces, sewage, and contaminated oysters. The number of reads obtained by MiSeq sequencing is shown at the top of the panel, and relative abundance of the sequence reads affiliated with a specific genotype of viruses is shown in the figure. The identified genotypes are indicated in the bars.

To validate the genotyping assay based on MFnPCR–MiSeq, viral RNA was extracted from two oyster samples (B005 and B006) in which the known NoV GII strains (i.e., NoV GII.4 Sydney 2012 and GII.17 Kawasaki 2014) were intentionally bioaccumulated, and the synthesized cDNA was subjected to MFnPCR–MiSeq. More than 73,115 sequence reads were identical to those of the artificially added NoV GII (**Figure 3**), thus, indicating the reliability of the genotyping assay by MFnPCR–MiSeq.

### Typing of Multiple Viruses in Clinical and Environmental Samples

MFnPCR–MiSeq was applied to eight human fecal and five sewage samples. Coinfection with strains NoV GI and NoV GII was identified in six fecal samples (**Figure 3**; 13K11602, 13K11801, 14K1604, 14K1702, 14K9202, and 14K9403). Genotyping of the detected NoV GI and GII was conducted by phylogenetic analysis of the MiSeq reads. All the sequence reads of NoV GI were found to be affiliated with GI.3 (**Figure 3**), whose sequence reads ended up in two phylogenetic clades (Supplementary Figure S1). The sequence reads from feces 13K11602 and 14K9202 were affiliated with clade I, while the other reads clustered in clade II. NoV GI.3 clade I and II contained the nucleotide sequence identified in the sewage sample from Nanning city, China, in 2011 (KM246914) and the sequence identified in a stool sample from Chinese adult outpatients in Beijing in 2007 (Accession No.: GQ856471), respectively. As for NoV GII, GII.4 (Supplementary Figure S2) was detected in fecal samples except for samples 14K1604 and 14K9704. GII.6 and GII.17 were identified in samples 14K1604 (Supplementary Figure S3) and 14K9704 (Supplementary Figure S4), respectively.

In the tested sewage samples collected at wastewater treatment plants in Niigata and Miyagi (designated as Niigata and Miyagi in **Figure 3**, respectively), NoV GII.3 (Supplementary Figure S5), NoV GII.4 (Supplementary Figure S2), NoV GII.6 (Supplementary Figure S3), SaV GII.3 (Supplementary Figure S6), and HAstV-1 (human AstV type 1), HAstV-2 (Supplementary Figure S7), and HAstV-MLB1 (Supplementary Figure S8) were detected (**Figure 3**). In the tested Niigata samples, concentrations of AiV (5.2 × 10^0^ to 1.7 × 10^1^ copies/μL), NoV GI (1.2 × 10^0^ to 1.7 × 10^1^ copies/μL), and EV (<7.8 × 10^1^ copies/μL) as determined by the MFqPCR assay (Kobayashi et al., 2017) were lower than the detection limit of MFnPCR–MiSeq as previously mentioned; therefore, those viruses were not detectable. On the other hand, SaV in samples Niigata_5_Jun, Niigata_24_Jul, and Niigata_5_Jan was not detectable although the concentrations were higher than 4.8 × 10^5^ copies/μL (Supplementary Table S2). As for the AstV in the Niigata samples, the dominant genotype changed with time, i.e., AstV was detected from May to July 2014, and the dominant genotype changed from HAstV-2 to HAstV-1.

## Discussion

Detection and genotyping of human pathogenic RNA viruses are essential for the identification of contamination sources, and for better understanding the circulation of those pathogens in the aquatic environments and human population. Here, we propose the MFnPCR–MiSeq assay, in which 11 RNA viruses are detected and quantified simultaneously on a microfluidic chip, and then genotyped by sequencing the recovered PCR amplicons. A single-tube multiplex PCR assay with subsequent agarose gel electrophoresis was previously developed to screen the RNA virus genotypes in fecal samples, but this assay has a number of limitations (Yan et al., 2003; Rohayem et al., 2004; Khamrin et al., 2011; Zeng et al., 2014). Firstly, primer sets yielding PCR amplicons with similar sequence lengths are not suitable because separation of such amplicons on an agarose gel is generally difficult. Secondly, nonspecific amplification can result in false positive detection of a virus. Finally, while analysis by cloning and Sanger sequencing is the accepted gold standard for genotyping of viruses (Nasri et al., 2007; Stene-Johansen et al., 2007; Kitajima et al., 2010), low-abundance viruses are likely to be overlooked if analysis has been carried out with an insufficient number of cloned DNA copies. In contrast, viruses were identified here by MFnPCR–MiSeq based on nucleic acid sequence similarity (and not on amplicon length), and sequence reads derived from nonspecific amplification were eliminated in blastn analysis. Our MiSeq sequencer yielded >20,000 reads per sample, allowing for the identification of low-abundance viruses (e.g., NoV GII.4 in the 13K11801 fecal sample).

Aside from PCR-based approaches, including our MFnPCR–MiSeq technique, shotgun metagenomic approaches have previously been used to detect human pathogenic viruses in sewage (Cantalupo et al., 2011; Ng et al., 2012), activated sludge (Tamaki et al., 2012; Bibby and Peccia, 2013), and reclaimed water (Rosario et al., 2009). A main advantage of metagenomic approaches is that they avoid bias introduced by PCR amplification of cDNA; i.e., PCR-based approaches require the use of oligonucleotide primers, with the consequence that viral populations with primer–template mismatches are overlooked. On the contrary, the population of human pathogenic viruses in environmental samples is generally small. When stool samples from patients with NoV infections were analyzed, even after enrichment of viral RNA by depletion of bacterial rRNA, the NoV reads accounted for only 0.01–1.9% of all sequence reads (Hasing et al., 2016). Therefore, metagenomic approaches require a massive sequencing effort per sample, which cannot be applied to multiple samples. Additionally, genotyping requires sequence information from specific genomic regions, such as the capsid N/S region of the NoV genome, and the probability of recovering sequence reads spanning those regions is low, especially when the targeted human pathogenic viruses form a low proportion of the total viral population. Shotgun metagenomic approaches should preferentially be used to study genetic diversity of whole viral populations. MFnPCR–MiSeq, on the contrary, enables us to perform reliable targeted genotyping of human pathogenic viruses with reasonable sensitivity (i.e., 10^1^–10^4^ copies/mL for sewage, 10^5^–10^8^ copies/g-human feces, and 10^2^–10^5^ copies/g-digestive tissues of oysters).

Notably, selection of appropriate oligonucleotide primers was critical for MFnPCR–MiSeq detection sensitivity because of variations in the amplification efficiency for each primer set, and also the possibility of primer–template mismatches resulting in false-negative detection of target viruses. In the present study, we could not detect SaV in the samples Niigata_5_Jun, Niigata_24_Jul, and Niigata_5_Jan even though the concentrations (4.8 × 10^5^ copies/μL) were higher than our method’s detection limit (10^3^ copies/μL for SaV). This result was probably a consequence of mismatches between the PCR primers and viral cDNA. SaV in the tested sewage samples was detectable by primers SaV1F, SaV5F, and SaV1245R, as well as the TaqMan probes, SaV124TP and SaV5TP, used in the previous assay version (MFqPCR), but was undetectable by the oligonucleotide primers selected in the present study (Supplementary Table S1). In addition to amplification efficiency and the possibility of primer–template mismatches, the length of the PCR amplicon should also be considered during selection of oligonucleotide primers for MFnPCR–MiSeq assay. This is due to the upper read-length limit restriction imposed by the Illumina MiSeq sequencer, which is typically 300 bp × 2 in a MiSeq paired-end sequencing reaction performed using the v3 reagent kit. Due to this limitation, we amplified and analyzed the capsid N/S region (∼350 nt) instead of the complete ORF2 (VP1) sequence (∼2,000 nt) of the NoV genome. Capsid N/S region sequences from environmental samples have provided important insights into NoV genotypes circulating in human society (Kazama et al., 2016), while classification of NoVs based on complete ORF2 (VP1) sequences (∼2,000 nt) has been proposed (Kroneman et al., 2013).

Source tracking of contaminating viruses requires investigation of a large number of samples. In clinical surveillance studies, for example, detection and genotyping of pathogenic viruses were carried out using more than 100 fecal samples (Román et al., 2003; Kageyama et al., 2004). Furthermore, NoV GII detection and genotyping was performed on 28 subsamples of frozen strawberries to identify the source of a contamination that caused a foodborne outbreak of gastroenteritis in Germany in 2012 (Mäde et al., 2013; Bernard et al., 2014). MFnPCR–MiSeq enables simultaneous detection and genotyping of 11 RNA viruses in up to 48 samples at once, making this a powerful tool for source tracking of human pathogenic RNA viruses. Additionally, as a microfluidic PCR technique has been applied for the detection and quantification of DNA viruses as well as RNA viruses (Ishii et al., 2014), with suitable modifications, our MFnPCR–MiSeq method can also be applied to detect DNA viruses. Moreover, recent advances in DNA sequencing techniques have accelerated the accumulation of viral sequence data, which has led to the development of novel PCR primer sets (Hata et al., 2015). These novel primer sets can be included in MFnPCR–MiSeq, along with the primers tested in the present study. MFnPCR–MiSeq should allow for further expansion of our understanding of the emergence and circulation of human pathogenic RNA viruses, and also DNA viruses, in natural environments, and human populations.

## Author Contributions

MO, TM, and SK performed the sample collection, MFnPCR–MiSeq analysis, and downstream bioinformatics analyses. All the authors wrote the manuscript, and reviewed and approved the final manuscript.

## Conflict of Interest Statement

The authors declare that the research was conducted in the absence of any commercial or financial relationships that could be construed as a potential conflict of interest.
