# Supplementary material for: Microfluidic PCR Amplification and MiSeq Amplicon Sequencing Techniques for High-Throughput Detection and Genotyping of Human Pathogenic RNA Viruses in Human Feces, Sewage, and Oysters
Source: Front Microbiol. 2018 Apr 27;9:830. doi: 10.3389/fmicb.2018.00830 (PMC5934477; doi:10.3389/fmicb.2018.00830)
Supplement: Supplementary file 1 [file Data_Sheet_1.docx]

Supplementary Material

**Microfluidic PCR amplification and MiSeq amplicon sequencing techniques for high-throughput detection and genotyping of human pathogenic RNA viruses in human feces, sewage, and oysters**

**Mamoru Oshiki^*^, Takayuki Miura, Shinobu Kazama, Takahiro Segawa, Satoshi Ishii, Masashi Hatamoto, Takashi Yamaguchi, Kengo Kubota, Akinori Iguchi, Tadashi Tagawa, Tsutomu Okubo, Shigeki Uemura, Hideki Harada, Naohiro Kobayashi, Nobuo Araki & Daisuke Sano**

*** Correspondence:** Mamoru Oshiki: oshiki@nagaoka-ct.ac.jp

#
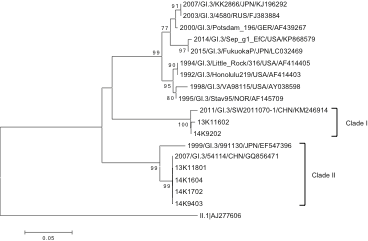
Supplementary Figure

# Figure S1. A neighbor-joining tree showing phylogenetic affiliation of norovirus (NoV) GI.3. The scale bar represents 5% sequence divergence.

**
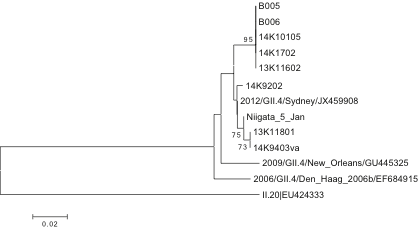
**

**Figure S2. A neighbor-joining tree showing phylogenetic affiliation of norovirus (NoV) GII.4.** The scale bar represents 2% sequence divergence.

**
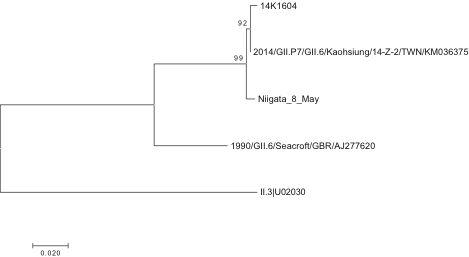
**

**Figure S3. A neighbor-joining tree showing phylogenetic affiliation of norovirus (NoV) GII.6.** The scale bar represents 2% sequence divergence.

**
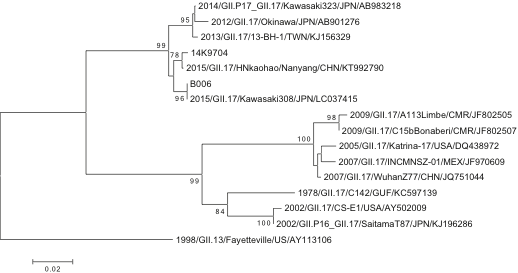
**

**Figure S4. A neighbor-joining tree showing phylogenetic affiliation of norovirus (NoV) GII.17.** The scale bar represents 2% sequence divergence.

**
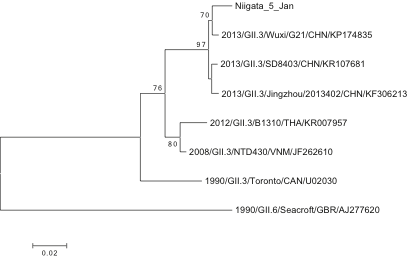
**

**Figure S5. A neighbor-joining tree showing phylogenetic affiliation of norovirus (NoV) GII.3.** The scale bar represents 2% sequence divergence.

**
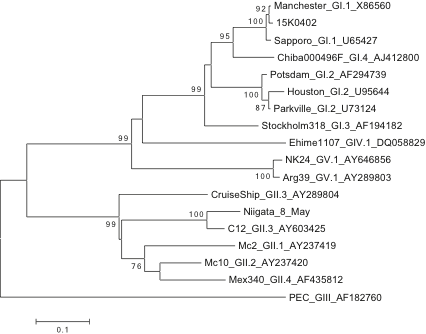
**

**Figure S6. A neighbor-joining tree showing phylogenetic affiliation of sapovirus (SaV).** The scale bar represents 10% sequence divergence.

**
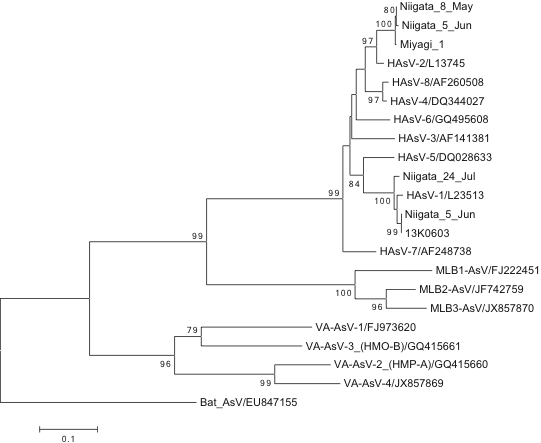
**

**Figure S7. A neighbor-joining tree showing phylogenetic affiliation of astrovirus (AsV)-HAstV.** The scale bar represents 10% sequence divergence.

**
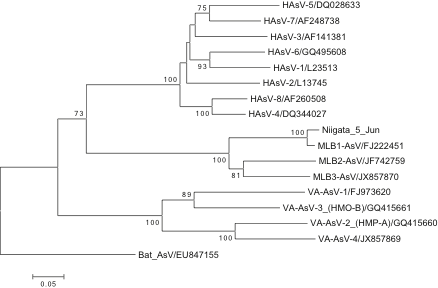
**

**Figure S8. A neighbor-joining tree showing phylogenetic affiliation of astrovirus (AsV)-HAstV-MLB.** The scale bar represents 5% sequence divergence.

# 2. Supplementary Table

**Table S1. Oligonucleotide primers used in this study.**

|  |  | 1st PCR | |  | Nested PCR | |  |  |  |
| --- | --- | --- | --- | --- | --- | --- | --- | --- | --- |
| Virus | Region | Primer^*^ | | Sequence (5' - 3') | Primer^*^ | | Sequence (5' - 3')^**^ | Size | Reference |
| AiV | VP1 | F | AiV-VP3-F1 | CACACCGCCCCTGCGTCRGCCCTCGT | F | AiV-VP1-F2 | CTCGATGCRCCMCAAGACACCGG | 504 | 1 |
|  |  | R | AiV-VP1-R1 | GAGAGCTGGAAGTCRAAGGG | R | AiV-VP1-R2 | CCTGACCAGTCCTCCCAWCCGAAGTA |  |  |
| AiV | VP1 | F | AiV-VP3-F1 | CACACCGCCCCTGCGTCRGCCCTCGT | F | AiV-VP1-F3 | GTGCTTCACRTACATCGCYGCGG | 238 | 1 |
|  |  | R | AiV-VP1-R1 | GAGAGCTGGAAGTCRAAGGG | R | AiV-VP1-R2 | CCTGACCAGTCCTCCCAWCCGAAGTA |  |  |
| AiV | 3C | F | Sense1(6261) | ACACTCCCACCTCCCGCCAGTA | F | Sense2 | GTACAAGGACATGCGGCG | 180 | 2 |
|  |  | R | Antisense 1 | AGGATGGGGTGGATRGGGGCAGAG | R | Antisense2 | CCTTCGAAGGTCGCGGCRCGGTA |  |  |
| AiV | 3CD-junktion | F | Sense1(6261) | ACACTCCCACCTCCCGCCAGTA | F | C94b | GACTTCCCCGGAGTCGTCGTCT | 223 | 3 |
|  |  | R | 6779 | GGAAGAGCTGGGTGTCAAGA | R | 246k | GACATCCGGTTGACGTTGAC |  |  |
| AstV | ORF1b | F | AHAstVF1 | AATCACTCCATGGGAAGCTCCT | F | AHAstVF2 | CAGAAGAGCAACTCCATCGCAT | 406 | 4 |
|  |  | R | AHAstVR1 | CCTARCGCYTGCACDGG | R | AHAstVR2 | GTRCTYCCWGTAGCRTCCTTAAC |  |  |
| EV | 5'UTR | F | Ev1F_66 | GGTACCYTTGTDYGCCTGTT | F | Ev1F_66 | GGTACCYTTGTDYGCCTGTT | 394 | This study |
|  |  | R | Ev1R_552 | CWCCCAAAGTAGTYGGTTYC | R | Ev2R_460 | CATTCAGGGGCCGGAGGA |  |  |
| EV | VP2 | F | AM11' | GARGCNTGYGGNTAYAGYGAYMG | F | AM21' | GGDTGGTGGTGGAARYTRCC | 367 | 5^†^ |
|  |  | R | AM31' | ARRTTWATDATYTGRTGNGG | R | AM31' | ARRTTWATDATYTGRTGNGG |  |  |
| NoV GI | capside | F | COG1F | CGYTGGATGCGNTTYCATGA | F | G1SKF | CTGCCCGAATTYGTAAATGA | 291 | 6 |
|  |  | R | G1-SKR | CCAACCCARCCATTRTACA | R | G1SKR | CCAACCCARCCATTRTACA |  |  |
| NoV GII | capside | F | COG2F | CARGARBCNATGTTYAGRT GGATGAG | F | G2SKF | CNTGGGAGGGCGATCGCAA | 301 | 6 |
|  |  | R | G2-SKR | CCRCCNGCATRHCCRTTRTACAT | R | G2SKR | CCRCCNGCATRHCCRTTRTACAT |  |  |
| NoV GIV | ORF2 | F | COG4F | TTTGAGTCYATGTAYAAGTGGATGC | F | G4SKF | CTTGGGAGGGGGATCGCGA | 343 | 7 |
|  |  | R | G4SKR | CCWCCAGCATATGARTTGTACAT | R | G4SKR | CCWCCAGCATATGARTTGTACAT |  |  |
| HAV | VP1-  capside | F | H1 | GGAAATGTCTCAGGTACTTTCTTTG | F | H3 | TCCTCAATTGTTGTGATAGC | 210 | 8 |
|  |  | R | H2 | GTTTTGCTCCTCTTTATCATGCTATG | R | H2 | GTTTTGCTCCTCTTTATCATGCTATG |  |  |
| HEV | ORF2 | F | 3156N | AATTATGCYCAGTAYCGRGTTG | F | 3158N | GTWATGCTYTGCATWCATGGCT | 348 | 9 |
|  |  | R | 3157N | CCCTTRTCYTGCTGMGCATTCTC | R | 3159N | AGCCGACGAAATCAA TTCTGTC |  |  |
| RV | VP7 | F | R1 | GGCTTTAAAAGAGAGAATTTCCGTCTGG | F | R3 | GTATGGTATTGAATATACCAC | 342 | 10 |
|  |  | R | R2 | GATCCTGTTGGCCATCC | R | R2 | GATCCTGTTGGCCATCC |  |  |
| RV | VP4 | F | con3 | TGGCTTCGCCATTTTATAGACA | F | con1_675 | YTRCCWCCRATTCARAAYAC | 212 | 11^†^ |
|  |  | R | con2_876 | ATTTCnGACCAYTTRTAWCC | R | con2_876 | ATTTCnGACCAYTTRTAWCC |  |  |
| SaV | RNA polymerase/  capside junction | F | SaV1245Rfwd | TAGTGTTTGARATGGAGGG | F | SaV1245Rfwd | TAGTGTTTGARATGGAGGG | 339 | 12 |
|  |  | R | SVR-DS3 | GGTGAVAVMCCATTYTCCAT | R | SVR-DS5 | CCCCACCCKGCCCACAT |  |  |
| SaV | RNA polymerase/  capside junction | F | SaV1245Rfwd | TAGTGTTTGARATGGAGGG | F | SaV1245Rfwd | TAGTGTTTGARATGGAGGG | 339 | 12 |
|  |  | R | SVR-DS4 | GGHGAHATNCCRTTBTSCAT | R | SVR-DS6 | CCCCAMCCMGCMMACAT |  |  |
| HPeV | VP1 | F | Cap-parEcho-F | TCHACWTGGATGMGRAARAC | F | VP1-parEchoF1 | CCAAAATTCRTGGGGTTC | 760 | 13 |
|  |  | R | Cap-parEcho-R | TCYARYTCACAYTCYTCYTC | R | VP1-parEchoR1 | AAACCYCTRTCTAAATAWGC |  |  |
| ^*^ F; forward primer, R; reverse, primer., ^**^ Universal tag was added to 5' end of forward and reverse primer;  forward; TCGTCGGCAGCGTCAGATGTGTATAAGAGACAG and reverse; GTCTCGTGGGCTCGGAGATGTGTATAAGAGACAG. ^†^: Primer sequences were slightly modified by adding degenerate bases. | | | | | | | | | |

**References**

1. Lodder, W.J., Rutjes, S.A., Takumi, K. and de Roda Husman, A.M. (2013) Aichi virus in sewage and surface water, the Netherlands. Emerg Infect Diseases 19: 1222-1230.
2. Oh, D.Y., Silva, P.A., Hauroeder, B., Diedrich, S., Cardoso, D.D.P. and Schreier, E. (2006) Molecular characterization of the first aichi viruses isolated in Europe and in South America. Arch. Virol. 151: 1199-1206.
3. Yamashita, T., Sugiyama, M., Tsuzuki, H., Sakae, K., Suzuki, Y. and Miyazaki, A.Y. (2000) Application of a reverse transcription-PCR for identification and differentiation of aichi virus, a new member of the picornavirus family associated with gastroenteritis in humans. J. Clin. Microbiol. 38: 2955-2961.
4. Hata, A., Katayama, H., Kitajima, M. and Furumai, H. (2015) Wastewater Analysis Indicates That Genetically Diverse Astroviruses, Including Strains Belonging to Novel Clades MLB and VA, Are Circulating within Japanese Populations. Appl. Environ. Microbiol. 81: 4932-4939.
5. Nasri, D., Bouslama, L., Omar, S., Saoudin, H., Bourlet, T., Aouni, M., Pozzetto, B. and Pillet, S. (2007) Typing of human enterovirus by partial sequencing of VP2. J. Clin. Microbiol. 45: 2370-2379.
6. Aw, T.G., Gin, K.Y.H., Oon, L.L.E., Chen, E.X. and Woo, C.H. (2009) Prevalence and genotypes of human noroviruses in tropical urban surface waters and clinical samples in Singapore. Appl. Environ. Microbiol. 75: 4984-4992.
7. Kitajima, M., Oka, T., Haramoto, E., Takeda, N., Katayama, K. and Katayama, H. (2010) Seasonal distribution and genetic diversity of genogroups I, II, and IV Noroviruses in the Tamagawa River, Japan. Environ. Sci. Technol. 44: 7116-7122.
8. Le Guyader, F., Dubois, E., Menard, D. and Pommepuy, M. (1994) Detection of hepatitis A virus, rotavirus, and enterovirus in naturally contaminated shellfish and sediment by reverse transcription-seminested PCR. Appl. Environ. Microbiol. 60: 3665-3671.
9. Huang, F.F., Haqshenas, G., Guenette, D.K., Halbur, P.G., Schommer, S.K., Pierson, F.W., Toth, T.E. and Meng, X.J. (2002) Detection by reverse transcription-PCR and genetic characterization of field isolates of swine hepatitis E virus from pigs in different geographic regions of the United States. J. Clin. Microbiol. 40: 1326-1332.
10. Le Guyader, F., Dubois, E., Menard, D. and Pommepuy, M. (1994) Detection of hepatitis A virus, rotavirus, and enterovirus in naturally contaminated shellfish and sediment by reverse transcription-seminested PCR. Appl. Environ. Microbiol. 60: 3665-3671.
11. Gentsch, J.R., Glass, R.I., Woods, P., Gouvea, V., Gorziglia, M., Flores, J., Das, B.K. and Bhan, M.K. (1992) Identification of group A rotavirus gene 4 types by polymerase chain reaction. J. Clin. Microbiol. 30: 1365-1373.
    Fischer, T.K., Page, N.A., Griffin, D.D., Eugen-Olsen, J., Pedersen, A.G., Valentiner-Branth, P., Mølbak, K., Sommerfelt, H. and Munk Nielsenf, N. (2003) Characterization of incompletely typed rotavirus strains from Guinea-Bissau: identification of G8 and G9 types and a high frequency of mixed infections. Virology 311: 125-133.
12. Sano, D., Pérez-Sautu, U., Guix, S., Pintó, R.M., Miura, T., Okabe, S. and Bosch, A. (2011) Quantification and genotyping of human sapoviruses in the Llobregat river catchment, Spain. Appl. Environ. Microbiol. 77: 1111-1114.
13. Pham, N.T.K., Trinh, Q.D., Khamrin, P., Maneekarn, N., Shimizu, H., Okitsu, S., Mizuguchi, M. and Ushijima, H. (2010) Diversity of human parechoviruses isolated from stool samples collected from Thai children with acute gastroenteritis. J. Clin. Microbiol. 48: 115-119.

**Table S2. Virus concentrations in the sewage samples.** The concentrations were determined previously by MFqPCR (Kobayashi et al., 2017). *n.d*.; not detected.

|  | Concentration (copies/µL) | |  |  |
| --- | --- | --- | --- | --- |
|  | Niigata_8_May | Niigata_5_Jun | Niigata_24_Jul | Niigata_5_Jan |
| AiV | 1.7 × 10^1^ | 5.2 × 10^0^ | 6.9 × 10^0^ | 5.5 × 10^1^ |
| AstV | 8.7. × 10^5^ | 5.1 × 10^6^ | 1.4 × 10^6^ | *n.d.* |
| EV | *n.d.* | 1.0 × 10^0^ | 7.8 × 10^1^ | *n.d.* |
| HAV | *n.d.* | *n.d.* | *n.d.* | *n.d.* |
| HEV | *n.d.* | *n.d.* | *n.d.* | *n.d.* |
| NoV GI | 4.9 × 10^0^ | 3.5 × 10^0^ | 1.2 × 10^0^ | 1.7 × 10^1^ |
| NoV GII | 1.3 × 10^1^ | 2.3 × 10^0^ | 2.6 × 10^1^ | 1.2 × 10^1^ |
| NoV GIV | *n.d.* | *n.d.* | *n.d.* | *n.d.* |
| RV | *n.d.* | *n.d.* | *n.d.* | *n.d.* |
| SaV | 1.5. × 10^6^ | 1.0 × 10^6^ | 4.8 × 10^5^ | 2.4 × 10^6^ |

**Reference:**

Kobayashi, N., Oshiki, M., Ito, T., Segawa, T., Hatamoto, M., Kato, T., Yamaguchi, T., Kubota, K., Takahashi, M., Iguchi, A., Tagawa, T., Okubo, T., Uemura, S., Harada, H., Motoyama, T., Araki, N. and Sano, D. (2017) Removal of human pathogenic viruses in a down-flow hanging sponge (DHS) reactor treating municipal wastewater and health risks associated with utilization of the effluent for agricultural irrigation. *Water Res.* 110, 389-398.**3. Supplementary Text**

*The development of oligonucleotide primers for detection of EV.*

In the present study, oligonucleotide primers Ev1F_66, Ev1R_552, and Ev2R_460 were newly designed for the detection of EV. For this purpose, all the available nucleic acid sequences affiliated with human enteroviruses A, B, C, and D (NCBI taxonomy IDs: 138948, 138949, 138950, and 138951, respectively) were downloaded from the National Center for Biotechnology Information website (11 November 2016). The 46,678 nucleic acid sequences were subjected to blastn analysis using the nucleic acid sequence corresponding to the conserved 5′ untranslated region (UTR) of an enterovirus as a reference sequence. The 6,482 nucleic acid sequences containing the 5′-UTR region were retrieved and were aligned in the ClustalW 1.83 software (Thompson et al., 1994). Gap opening and extension penalties in a pairwise alignment were set to 10 and 0.1, respectively. Conserved regions were examined manually, and oligonucleotide primers were designed and synthesized by Eurofin Genomics (Tokyo, Japan).
